# A novel fusion based on the evolutionary features for protein fold recognition using support vector machines

**DOI:** 10.1038/s41598-020-71172-x

**Published:** 2020-09-01

**Authors:** Mohammad Saleh Refahi, A. Mir, Jalal A. Nasiri

**Affiliations:** 1 Department of Electrical Engineering, Amirkabir University of Technology, Tehran, Iran; 2 Iranian Research Institute for Information Science and Technology (IranDoc), Tehran, Iran

**Keywords:** Classification and taxonomy, Protein folding

## Abstract

Protein fold recognition plays a crucial role in discovering three-dimensional structure of proteins and protein functions. Several approaches have been employed for the prediction of protein folds. Some of these approaches are based on extracting features from protein sequences and using a strong classifier. Feature extraction techniques generally utilize syntactical-based information, evolutionary-based information and physicochemical-based information to extract features. In recent years, finding an efficient technique for integrating discriminate features have been received advancing attention. In this study, we integrate Auto-Cross-Covariance and Separated dimer evolutionary feature extraction methods. The results’ features are scored by Information gain to define and select several discriminated features. According to three benchmark datasets, DD, RDD ,and EDD, the results of the support vector machine show more than 6$\%$ improvement in accuracy on these benchmark datasets.

## Introduction

Proteins are Jack of all trades biological macromolecules. They are involved in almost every biological reaction; Protein plays a critical role in many different areas such as building muscle, hormone production, enzyme, immune function, and energy. Typically more than 20,000 proteins exist in human cells^1^, to acquire knowledge about the protein function and interactions, the prediction of protein structural classes is extremely useful^2^. Fold recognition is one of the fundamental methods in protein structure and function prediction.

Each type of protein has a particular three-dimensional structure, which is determined by the order of the amino acids in its polypeptide chain. A protein’ s structure begins with its amino acid sequence, which is thus considered its primary structure. The next level of the organization includes the $\alpha$ helix and $\beta$ sheets that forms with certain segments of the polypeptide chain; these folds are elements of secondary structure. The full, three-dimensional conformation formed by an entire polypeptide chain is referred to as tertiary structure^3^. One of the main steps which can be assumed as a vital stage for predicting protein fold(secondary structure) is feature extraction. Computational feature extraction methods are divided into syntactical, physicochemical and evolutionary methods. Syntactical methods pay attention only to the protein sequence, like composition and occurrence^4–6^. physicochemical methods consider some physical and chemical properties of protein sequences. Evolutionary methods extract features from Basic Local Alignment Search Tool(BLAST).

When attempting to solve many biological problems, it is obvious that a single data source might not be informative, and combining several complementary biological data sources will lead to a more accurate result. When we studied methods of protein fold recognition, we found that less attention has been paid to the fusion of features to get more comprehensive features. In recent studies, researchers attempted to find new feature extraction methods^7–12^ or train different classifiers to achieve high accuracy^13–18^, even though some problems like incomplete data sources, false positive information, multiple aspect problem, and so on encourage us to combine data sources.

Hence, to prepare more informative and discriminative features, we use Auto-Cross-Covariance(ACC)^10^ and Separated dimer(SD)^9^ methods. Because SD explores some amino acid dimers that may be non-adjacent in sequence^9^ and ACC method measures the correlation between the same and different properties of amino acids^10^. One of the main advantages of ACC and SD is to find a fixed length vector from a variable protein length. The performance of the proposed method is evaluated using three benchmark datasets DD^4^ , RDD^19^ and EDD^10^.

In this paper, we focus on fusing ACC and SD feature extraction methods based on Position Specific Scoring Matrix(PSSM) generated by using the Position-Specific Iterated BLAST(PSI-BLAST) profile to predict protein fold. The 1600 ACC features and the 400 SD features are extracted based on the PSSM. Finally, we construct a reduced-dimensional feature vector for the Support Vector Machine (SVM) classifier by using the Information Gain(IG).

## Background

In 1997, Dubchak et al. studied syntactical and physicochemical method^20^. In which they assumed five properties of amino acid such as hydrophobicity (H), frequency of $\alpha$ helix (X), polarity (P), polarizability (Z) and van der Waals volume (V). Recently a novel fusion approach called Forward Consecutive Search (FCS)^21^ scheme that combined physicochemical-based by syntactical-based features. Then Enhanced Artificial Neural Network trained on benchmark datasets for obtaining high accuracy in protein fold recognition. In 2009, pairwise frequencies of amino acids separated by one residue (PF1) and pairwise frequencies of adjacent amino acid residues (PF2) were proposed by Ghatny and Pal^7^. Taguchi and Gromiha^5^ have proposed features that are based on the amino acid occurrence.

Another solution to find similarity between protein sequences is based on the BLAST. Many feature extraction methods utilize BLAST alignments to extract the possibility of amino acid in specific positions called PSSM.The bigram feature extraction method was introduced by Sharma et al.^8^ that the related feature vector was computed by counting the bigram frequencies of occurrence from PSSM. This represented the transitional probabilities from one amino acid to another and also produces 400 features. Lyons et al.^22^ employed the HMMâ€“HMM alignments of protein sequence from HHblits to extract the profile HMM (PHMM) matrix. They computed the distances between several PHMM matrices to find the alignment path using dynamic programming. If the distance matrix between two proteins was low, they belonged to the same fold otherwise they did not. An innovative predictor called PFPA containing an ensemble learning classifier and a novel feature set that combined the information from PSI-BLAST^23^. In 2011, the AAC-PSSM-AC method was proposed by Liu et al.^24^. This method combined PSSM with Auto Covariance (AC)transformation to extract features, and the prediction accuracy reached about 74% in both datasets 25PDB and 1189. The different technique recommended as a feature extraction method was separated dimers(SD)^9^ which were used the probabilistic expressions of amino acid dimer occurrence that had varying degrees of spatial separation in protein sequences. Dong et al.^10^ proposed autocross-covariance (ACC) transformation for protein fold recognition. ACC could measure the correlation of two properties along the protein sequence and transform the matrix into a fixed-length vector. A novel TSVM-fold employed a group of pairwise sequence similarity scores created by HHblits, SPARKS-X, and DeepFR template-based methods. The results’ features of the attributes of the sequences were applied to the SVM for the protein fold recognition^25^. A big data feature selection method based on the Map-Reduce framework and Vortex Search Algorithm (VSA) was introduced by Jazayeri et al.^26^ , which had considerable prediction accuracy in protein fold recognition. Moreover, Pailwal et al.^11^ proposed the ability of trigram to extract features from the neighborhood information of amino acid.

In addition to the feature extraction methods, some researchers have paid attention to classification methods for protein fold recognition. In^13^ Kohonen’s selfâ€“organization neural network was used and showed the structural class of protein was considerably correlated with its amino acid composition features. Baldi et al.^27^ employed Recurrent and Recursive Artificial Neural Networks (RNNs) and mixed it by directed acyclic graphs (DAGs) to predict protein structure. In^15^, classwise optimized feature sets were used and SVM classifiers were coupled with probability estimates to make the final prediction. Linear discriminant analysis(LDA) was employed to evaluate the contribution of sequence parameters in determining the protein structural class. Parameters were used as inputs of the artificial neural networks^28^. The composition entropy was proposed to represent apoptosis protein sequences, and an ensemble classifier FKNN (fuzzy K-nearest neighbor) was used as a predictor^16^. TAXFOLD^29^method extracted sequence evolution features from PSI-BLAST profiles and also the secondary structure features from PSIPRED profiles, finally a set of 137 features is constructed to predict protein folds. Sequence-Based Prediction of Proteinâ€“Peptide(SPRINT) method was used to the prediction of Proteinâ€“peptide Residue-level Interactions by SVM^14^. SVM implements the structural risk minimization (SRM) that minimized the upper bound of generation error^30,31^. Jones et al.^32^ suggested the DeepCov method which employed convolutional neural networks to operate on amino acid pair frequency and covariance data that extract from sequence alignments. DeepSFâ€”a deep learning method of classifying protein sequences into folds was also employed to identify templates for the target^33^. In^34^ was attempted to show Artificial Neural Network (ANN) with different feature extraction method was more accurate than other classifier methods. In another study, Gosh et al.^35^ proposed a two-stage framework for feature extraction and classification. They utilized sequence-based and structure-based features in their framework which removed redundant features by, mutual information (MI) feature selection method. At the final, a boosting classifier based on Random Forest, K-nearest neighbor (KNN), and multi-layer perceptron (MLP) show the considerable result in prediction accuracies.

## Methods

**Figure 1:**
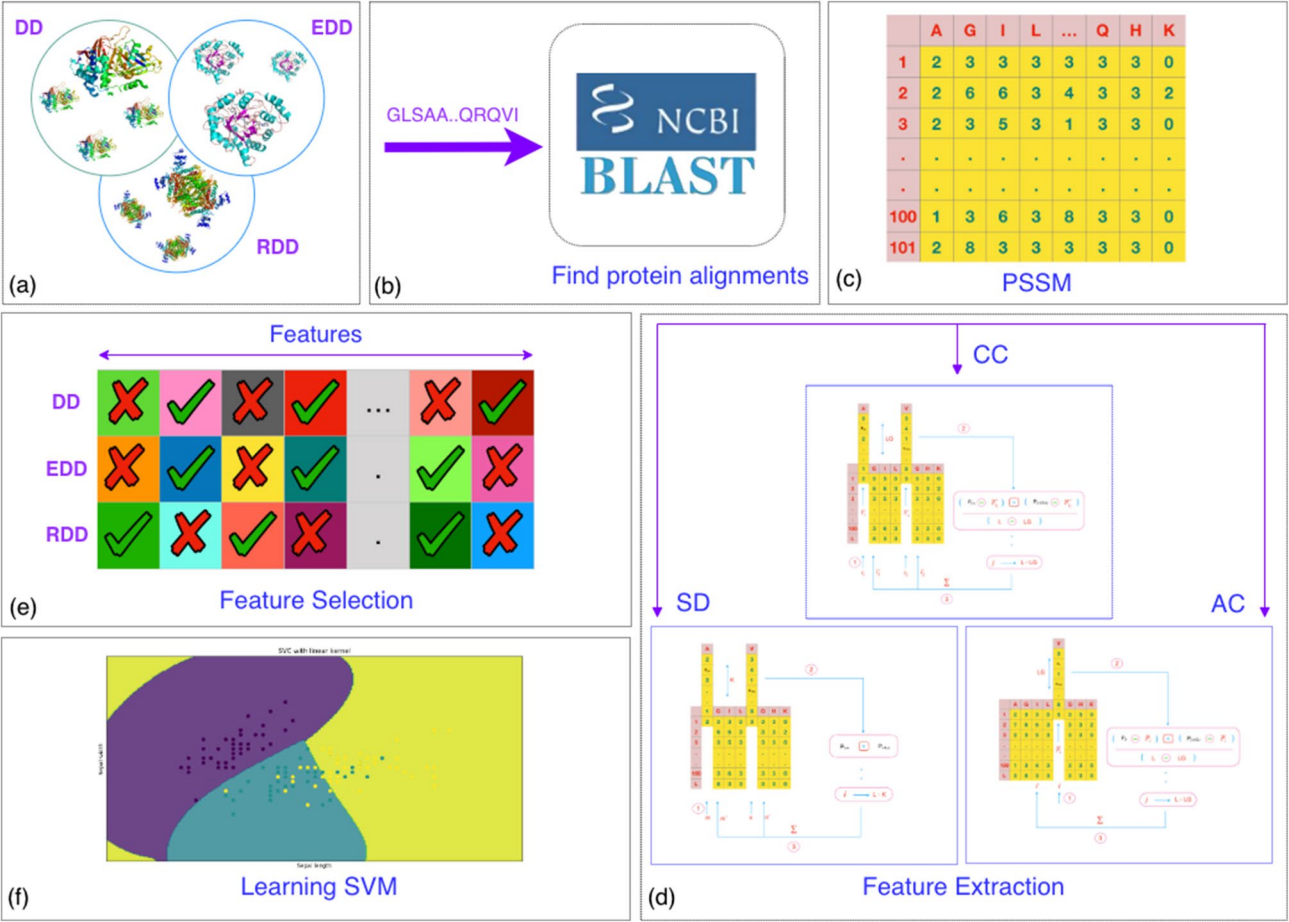
Illustrates the framework of proposed protein fold recognition method. (**a**) Using protein sequences of three benchmark datasets. (**b**) Sequence alignments are found for each protein sequence by BLAST. (**c**) PSSM is calculated for each protein. (**d**) ACC and SD methods are used to extract features from PSSM. (**e**) The features are selected by IG. (**f**) SVM algorithm is trained to classify proteins.

This section illustrates the step-by-step of the proposed method for protein fold recognition. In the first step, sequence alignments are found for each protein using BLAST. To show improvements in protein fold recognition using evolutionary information that are presented in PSSM(Preprocessing), therefore ACC^10^ and SD^9^ features are extracted from PSSM(Feature extraction). In the next step, the features are combined and then selected by the IG. In the last step, the SVM algorithm is trained to classify proteins. A comprehensive view of this approach can be found in Fig. 1.

### Preprocessing

#### BLAST

Similarity is used here to mention the resemblance or percentage of identity between two protein sequences^36^. The similarity search depends on the bioinformatics algorithm. Basic Local Alignment Search Tool(BLAST) is a tool that helps researchers to compare a query sequence with a database of sequences and identify specific sequences that resemble the query sequence above a certain threshold. BLAST is a local alignment algorithm that means to find the region (or regions) of the highest similarity between two sequences and build the alignment outward from there^37^.

#### PSSM

Position Specific Scoring Matrix(PSSM) is applied to express motif in a protein sequence. P-BLAST searches in which amino acid substitution scores are given separately for each position in a protein multiple sequence alignment. In this paper, PSSM is used to extract features by ACC and SD methods.

### Feature extraction

#### ACC

ACC fold^10^ utilizes autocross-covariance transformation that convert the PSSMs of different lengths into fixed-length vectors. The ACC is separated into two kinds of features: AC between the same properties, cross-covariance (CC) between two different properties. The AC variable measures the correlation of the same property between two properties separated by LG, distance along the sequence:

1 $$\begin{aligned} AC(i,LG)=\sum _{j=1}^{L-LG} (P_{j,i}-\overline{P_i})(P_{j+LG,i}-\overline{P_i})\setminus {(L-LG)} \end{aligned}$$

where $P_{j,i}$ is the PSSM score of amino acid *i* at position *j*,and $\overline{P_i}= \sum _{j=1}^{L} P_{j,i}\setminus {L}$, the average score of an amino acid *i* in the total protein sequence. The number of features which are calculated from AC is $20\times LG$. The CC measures the correlation of two different properties between the distances of LG along the sequence:

2 $$\begin{aligned} CC(i_1,i_2,LG)=\sum _{j=1}^{L-LG} (P_{j,i_1}-\overline{P_{i_1}})(P_{j+LG,i_2}-\overline{P_{i_2}})\setminus {(L-LG)} \end{aligned}$$

where *i*_1_,*i*_2_ are two different amino acids and $\overline{P_{i1}}(\overline{P_{i2}})$is the average score for amino acid *i*1 (*i*2) along the sequence. The CC variables are not symmetric. The total number of CC variables is $380 \times LG$.The combination of AC and CC features make $400 \times LG$ feature vectors.

#### SD

Separated Dimer(SD) method was introduced by Saini et al.^9^. It is employed to extract features from amino acids that may or may not be adjacent in the protein sequence. The SD demonstrates the probabilities of the occurrence of amino acid. SD generates 400 features.

3 $$\begin{aligned} F(k)= [F_{1,1}(k),F_{1,2}(k),\ldots ,F_{20,19}(k),F_{20,20}(k)] \end{aligned}$$

*F*(*k*) is computed as the feature sets for probabilistic occurrence of amino acid dimers with different values of *k* which is a special distance between dimers. *P* represents the PSSM matrix for a protein sequences. It is $L \times 20$ matrix where L is the length of the protein sequence:

4 $$\begin{aligned} F_{m,n}(k)= \sum _{i=0}^{L-k} P_{i,m} P_{i+k,n} \end{aligned}$$

in which *m*, *n* ($1 \le m , n \le 20$) are the scores of two selective amino acids in PSSM.

### Fusion hypothesis

**Figure 2:**
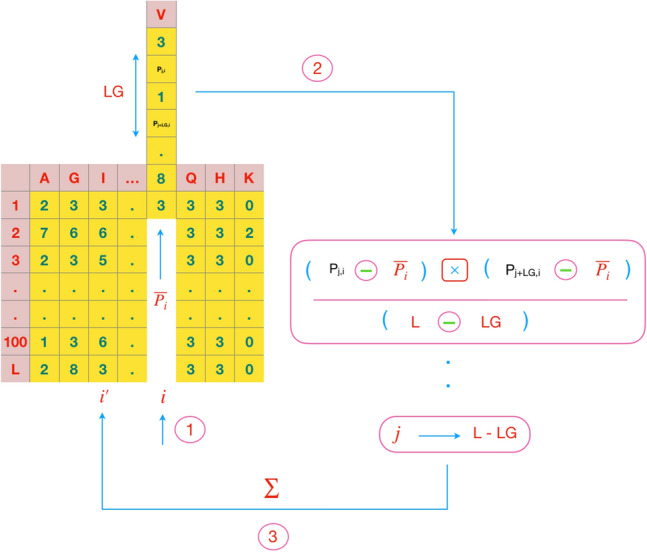
The AC features of the ACC, is measured by the correlation of the same property between two properties separated by a distance of LG along the sequence.

**Figure 3:**
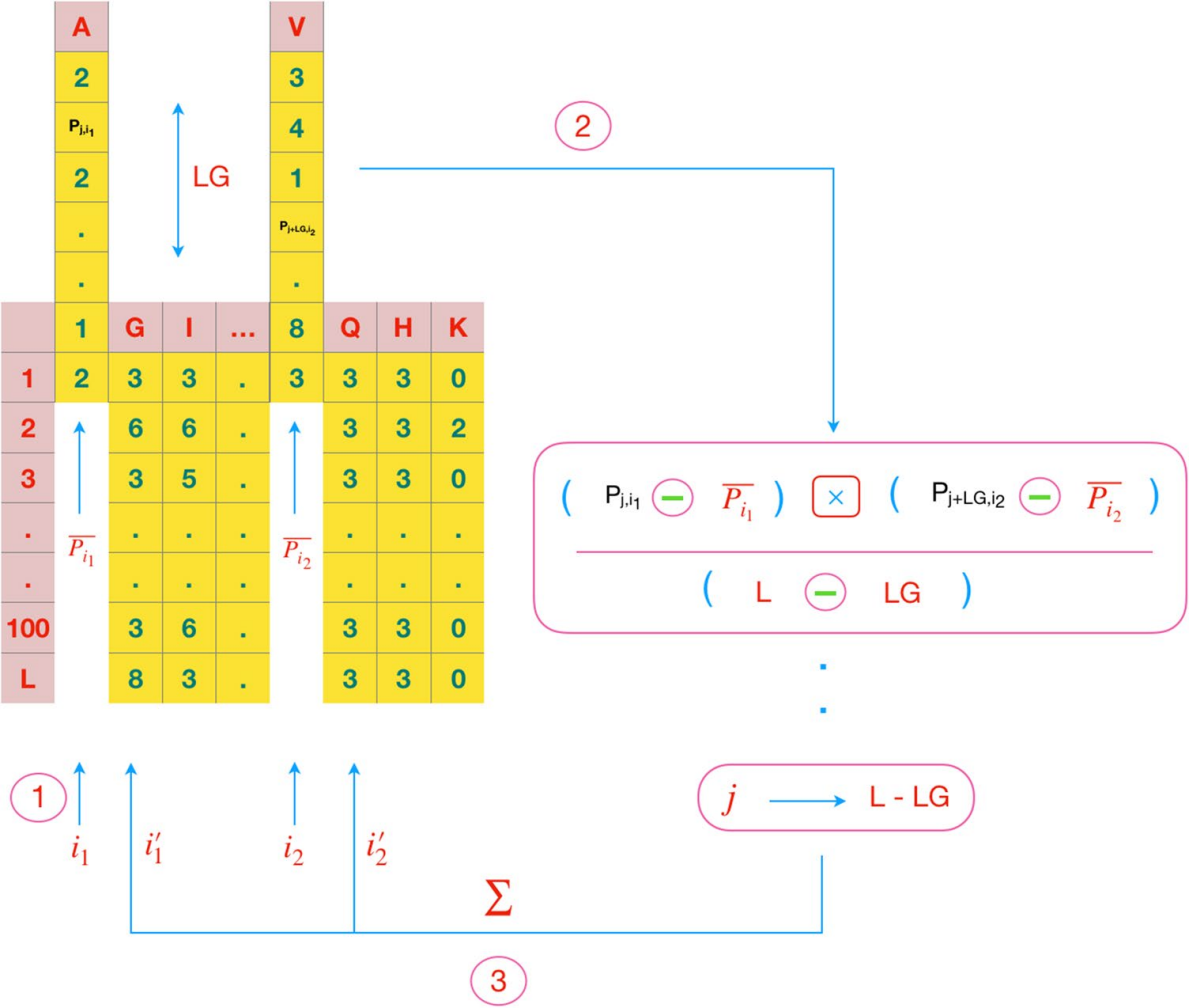
The CC features of the ACC,is measured by the correlation of two different properties between the distances of LG along the sequence.

**Figure 4:**
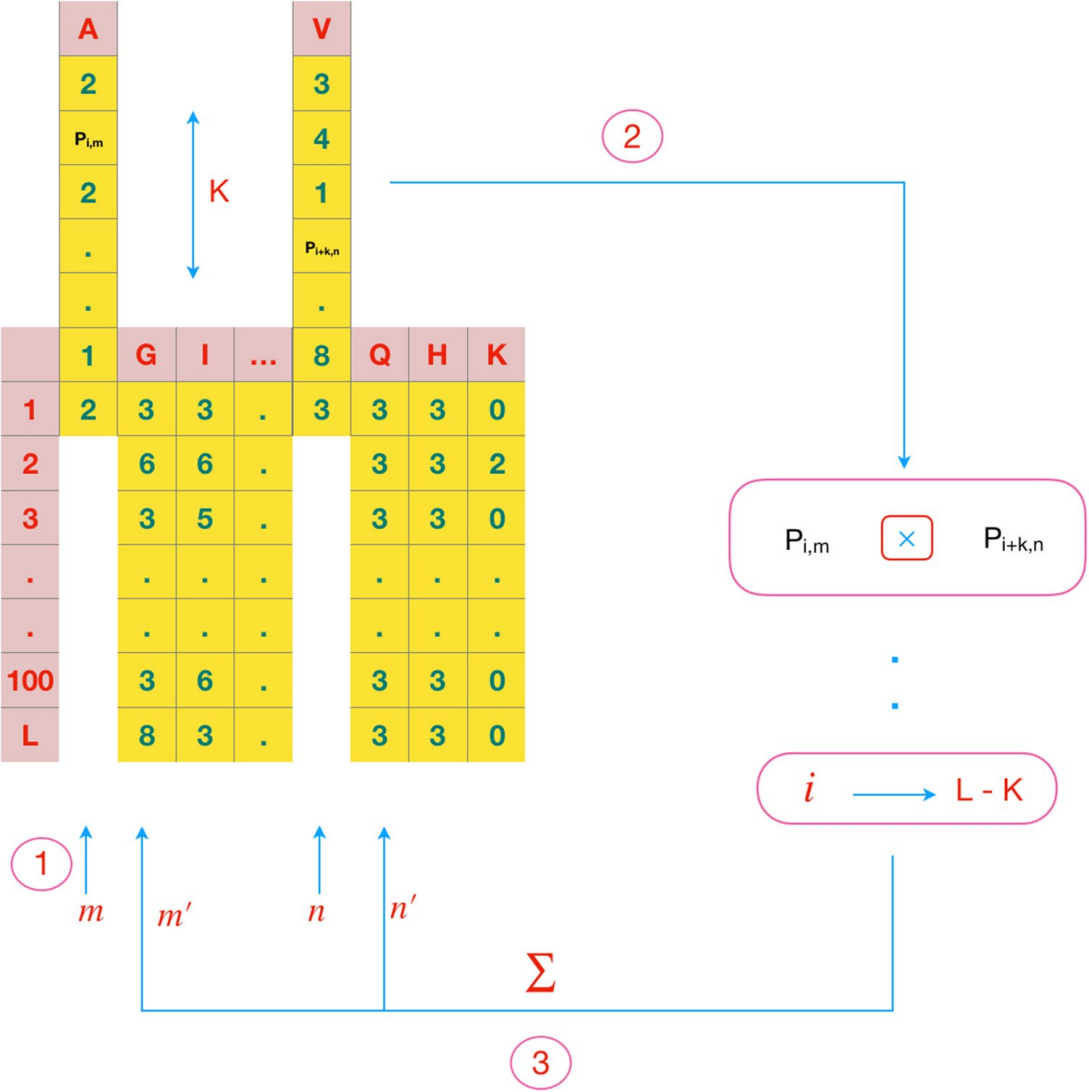
The SD consist of amino acid dimers with probabilistic expressions that have k separation.

More attention needs to be paid to find an efficient technique for integrating distinct data sources for the protein fold recognition problem^38^. Various techniques have been employed based on the features which are extracted from protein sequences. These techniques investigate different aspects of a sequence like the study of possible position of amino acids, protein chemical characteristics, syntactical features, and so on. Hence, integrating them can model the folding problem more accurate. In this study, three hypotheses have been considered for fusion data sources. The first, only evolutionary features are used since integrating different types of features may have an undesirable effect on each other. According to our studies, the results of ACC and SD methods are correlated. Recent papers showed that there are two behaviors for the recall and precision of each fold. First, the recall (precision) of both methods can be high; second, if the recall(precision) of one method(ACC) is less, then the recall(precision) of the other method(SD) is high. So, ACC and SD can be the complement of each other and these behaviors can be seen in almost every fold. Hence, the next hypothesis is the choice of ACC and SD. The last hypothesis is that ACC and SD features exhibit a relationship between amino acids which may or may not be adjacent. In this approach, three different characters are defined which show each amino acid in a specific position what relation has with others. These characters are shown in Figs. 2, 3 and 4.

### Feature selection

#### Information gain

Feature selection is a common stage in classification problems. It can improve the prediction accuracy of classifiers by identifying relevant features. Moreover, feature selection often reduces the training time of a classifier by reducing the number of features which are going to be analyzed. Information gain (IG) is a popular feature selection method. It ranks features by considering their presence and absence in each class^39^. The IG method gives a high score to the features that occur frequently in a class and rarely in other classes^40^. For any variable *X* from the features, its information entropy is determined:

5 $$\begin{aligned} I(X)=-\sum \limits _{i}{P(x_i)log_{2}(P(x_i))} \end{aligned}$$

$x_i$ denotes a set of values of *X*, and $P(x_i)$ expresses the prior probability of $x_i$. The conditional entropy of *X* under the condition of *Y* is defined as:

6 $$\begin{aligned} I(X|Y)=-\sum \limits _{j}{P(y_j)}\sum \limits _{i}{P(x_i|y_i)log_{2}(P(x_i|y_i))} \end{aligned}$$

where $P(x_i|y_j)$ is the posterior probability of $x_i$ given the value $y_j$ of *Y*. Then, information gain *IG*(*X*|*Y*) is calculated by:

7 $$\begin{aligned} IG(X|Y)=I(X)-I(X|Y) \end{aligned}$$

### Support vector machine

Support Vector Machine (SVM) was proposed by Vapnik and Cortes^41^. It is a powerful tool for binary classification. SVM is on the basis of Structural Risk Minimization (SRM) and Vapnik-Chervonenkis (VC) dimension. The central idea of SVM is to find the optimal separating hyperplane with the largest margin between the classes. Due to the SRM principle, SVM has great generalization ability. Moreover, the parameters of the optimal separating hyperplane can be obtained by solving a convex quadratic programming problem (QPP), which is defined as follows:

8 $$\begin{aligned} \begin{aligned} \min _{w} \quad&\frac{1}{2}{{\left\| w \right\| }^{2}}+C\sum \limits _{i=1}^{n}{{{\xi }_{i}}} \\ \text {s.t. } \quad&{{y}_{i}}({{w}^{T}}{{x}_{i}}+b)\text { }+{{\xi }_{i}}\ge 1,\forall i \end{aligned} \end{aligned}$$

where $\xi$ is the slack variable associated with $x_{i}$ sample and *C* is a penalty parameter. Note that the optimization problem can be solved when the classification task is linearly separable. In the case of nonlinear problems, the input data is transformed into a higher-dimensional feature space in order to make data linearly separable. It makes possible to find a nonlinear decision boundary without computing the parameters of the optimal hyperplane in a high dimensional feature space^42^.

As mentioned in this subsection, SVM is designed to solve binary classification problems. However, there are multi-class approaches such as One-vs-One (OVO) and One-vs-All (OVA)^43^, which can be used for solving multi-class classification problems. In this paper, we used OVO strategy.

### Dataset

Three popular datasets are employed in this study, are DD dataset^4^, EDD dataset^10^, and RDD dataset^19^. DD dataset contains 27 folds which represent four major structure classes: $\alpha$, $\beta$, $\frac{\alpha }{\beta }$, $\alpha + \beta$. The training set and the testing set contain 311 and 383 sequences respectively, whose sequence similarity is less than $35\%$^4^. The EDD dataset consists of 3418 proteins with less than $40\%$ sequential similarity belonging to the 27 folds that originally are adopted from the DD dataset. The RDD dataset consists of 311 protein sequences in the training and 380 protein sequences in testing datasets with a similarity lower than 37%^19^.

**Figure 5:**
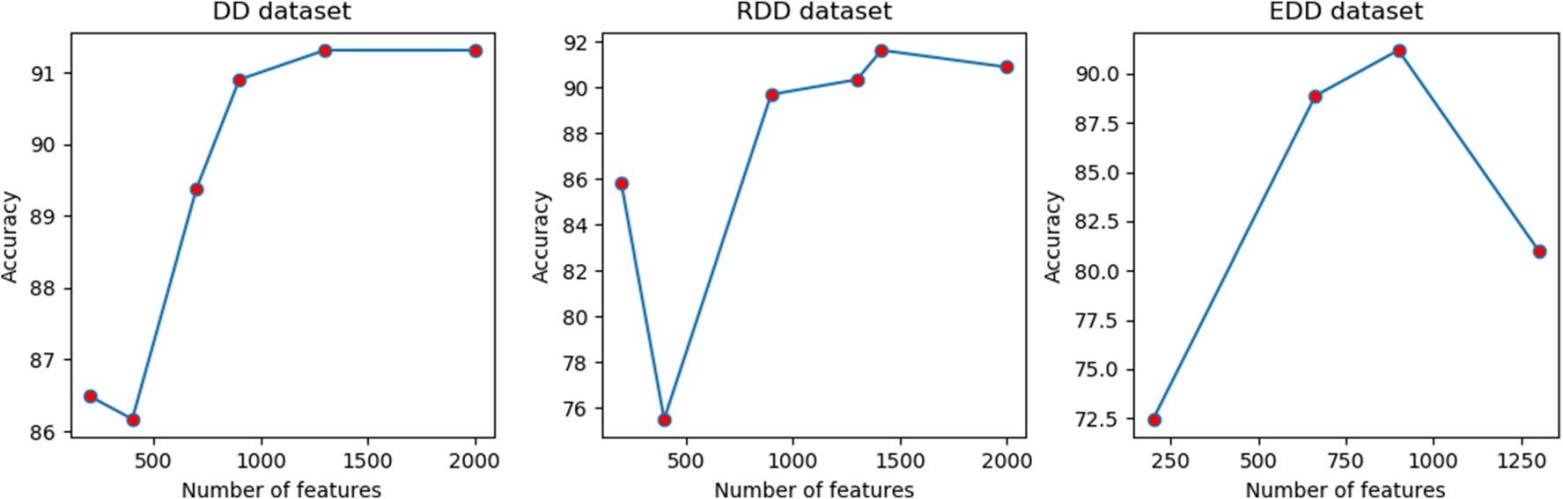
Comparison of number of features and accuracy for DD, RDD and EDD datasets to evaluate the IG method.

### Performance measures

This research employs performance measures such as sensitivity, precision, and F1 Score to produce various statistical results. The first of them is Sensitivity that measures the ratio of correctly classified samples to the whole number of test samples for each class which is classified as correct samples and calculated as follows:

9 $$\begin{aligned} Sensitivity=\frac{TP}{TP+FN}\times 100 \end{aligned}$$

TP represents true positive and FN represents false negative samples. Precision represents, how relevant the number of TP is to the whole number of positive prediction and is calculated as follows:

10 $$\begin{aligned} Precision=\frac{TP}{TP+FP}\times 100 \end{aligned}$$

FP denotes false positive. F1 Score is the weighted average of Precision and Recall. F1 score, as other evaluation criteria which are used in this study measures, is calculated as follows:

11 $$\begin{aligned} F1 score=\frac{2TP}{2TP+FP+FN}\times 100 \end{aligned}$$

## Results

### Classification and hyper-parameter tuning

The experiments are performed on the benchmark datasets to evaluate the performance of the classification. we also utilize the 10-fold cross-validation in this study, which has done by many researchers to examine predictive potency. In this study, LibSVM^44^ with RBF (Radial Basis Function) as the kernel functions has been used. The C parameter is optimized by search between $\{2^{-14},2^{-13},\dots ,2^{13},2^{14}\}$ and also $\Gamma$ parameter of RBF is considered between $\{2^{-14},2^{-13},\dots ,2^{13},2^{14}\}$. The SVM is originally designed for binary data classification. This study use OVO method to approach a multi-class classifier.

### Feature engineering

The details of the feature extraction method are explained in methodology, but it is important to know how far is assumed between aminoacids, for each ACC and SD methods. In developing the algorithm to extract features from PSSM, *LG* and *k* parameters have been assumed like ACC and SD papers values^9,10^. We consider both *LG* and *k* equals to 4. So the final number of features for ACC are 1600 features and the number of features of SD are 400. The IG^39^ makes our method safe from noisy features. In this approach, the features which are ranked between $[\frac{1}{2}\max _{IG} ,\max _{IG} ]$, are determined for each dataset. The results of IG for each dataset are exhibited in Table 1.

## Discussion

**Figure 6:**
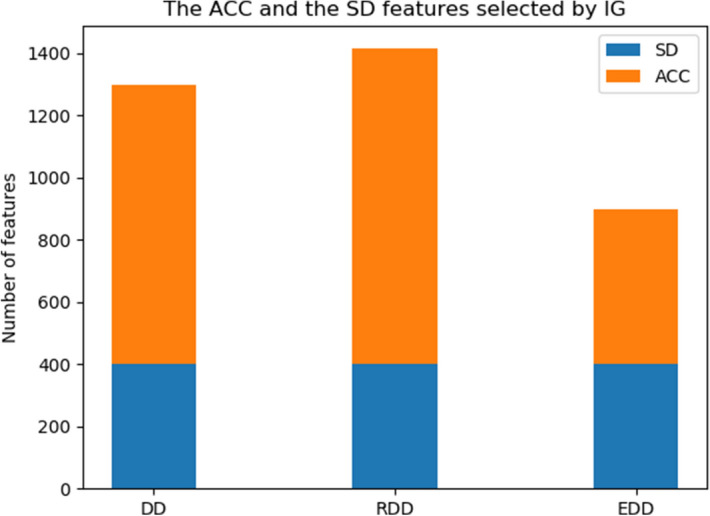
Comparison of the ACC and the SD in DD, RDD and EDD datasets.

Table 2 illustrates the total prediction accuracies of the existing approaches for classification of protein folds in the DD,RDD and EDD datasets. Table 2 also shows the success rates of our proposed fusion approach. According to Table 2, classification results of the combined ACC and SD followed by selection of best features by IG show considerable improvement compared to the state of art. Enhanced-SD has been exhibited quite promising results on DD and EDD datasets. ACC, SD and PFPA feature sets are also giving quite promising results on the three datasets in comparison with the other feature extraction methods. For the EDD dataset, the Enhanced-SD features reach 93% recognition accuracy. Our proposed method gives the best recognition performance for the other datasets. For the DD dataset, it is giving 91.31% recognition accuracy. For the RDD and EDD datasets, the recognition accuracies are 91.64% and 91.2%. Our results are on average around 5%, 8% and 14% better than the Enhanced-SD, SD, and ACC respectively. This is a significant improvement in terms of recognition accuracy when compared with existing feature extraction techniques. Figure 6 has been shown to figure out the result distribution of feature selection method. Even though the number of ACC in the three datasets are more, but all of the SD features exist in the selected features. However, we study and compare SD and ACC methods separately, we find out that the fusion of them can make more informative data which cover all characteristics of folds.

**Figure 7:**
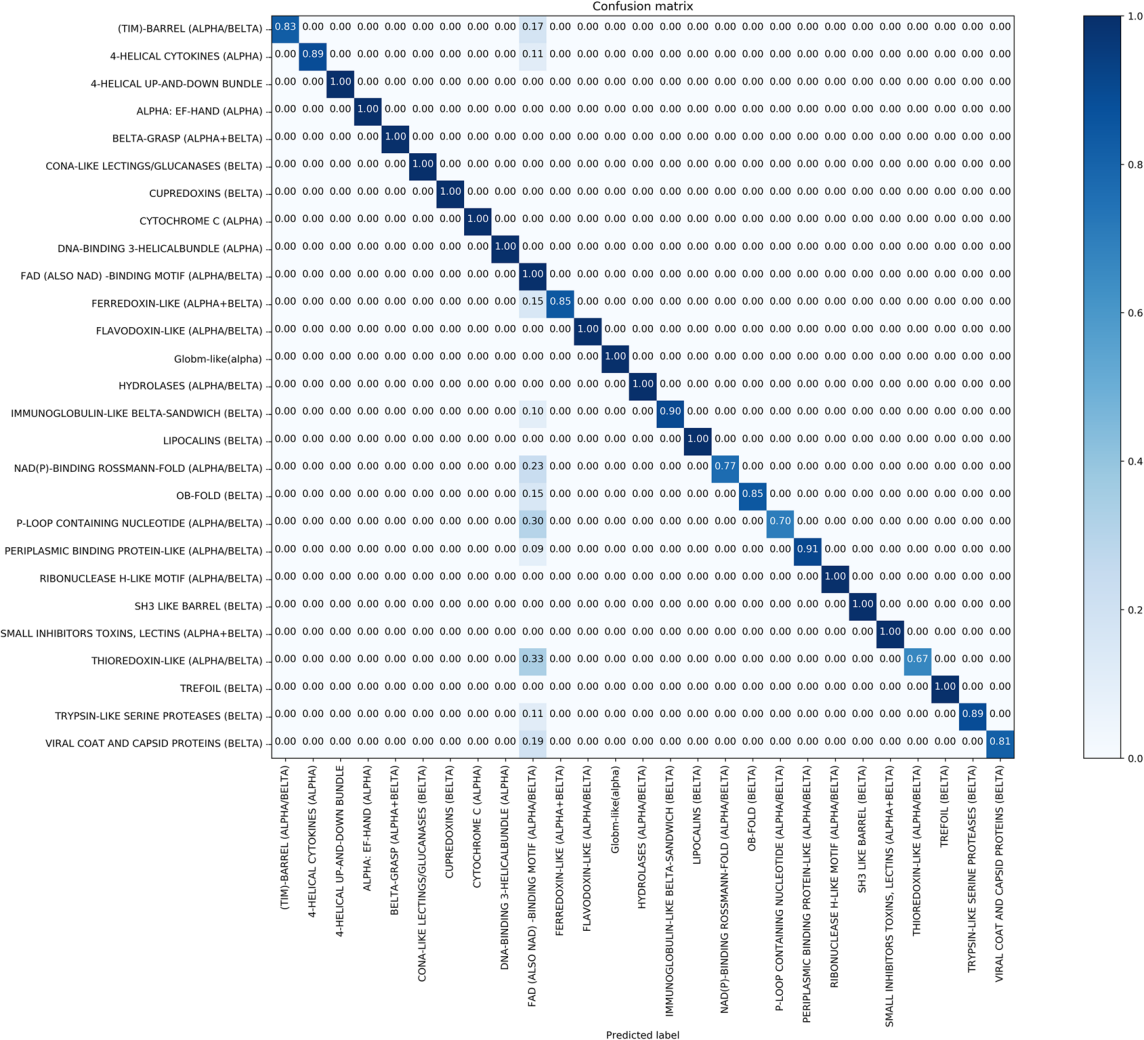
Confusion matrix of DD dataset ($91.31\%$).

**Figure 8:**
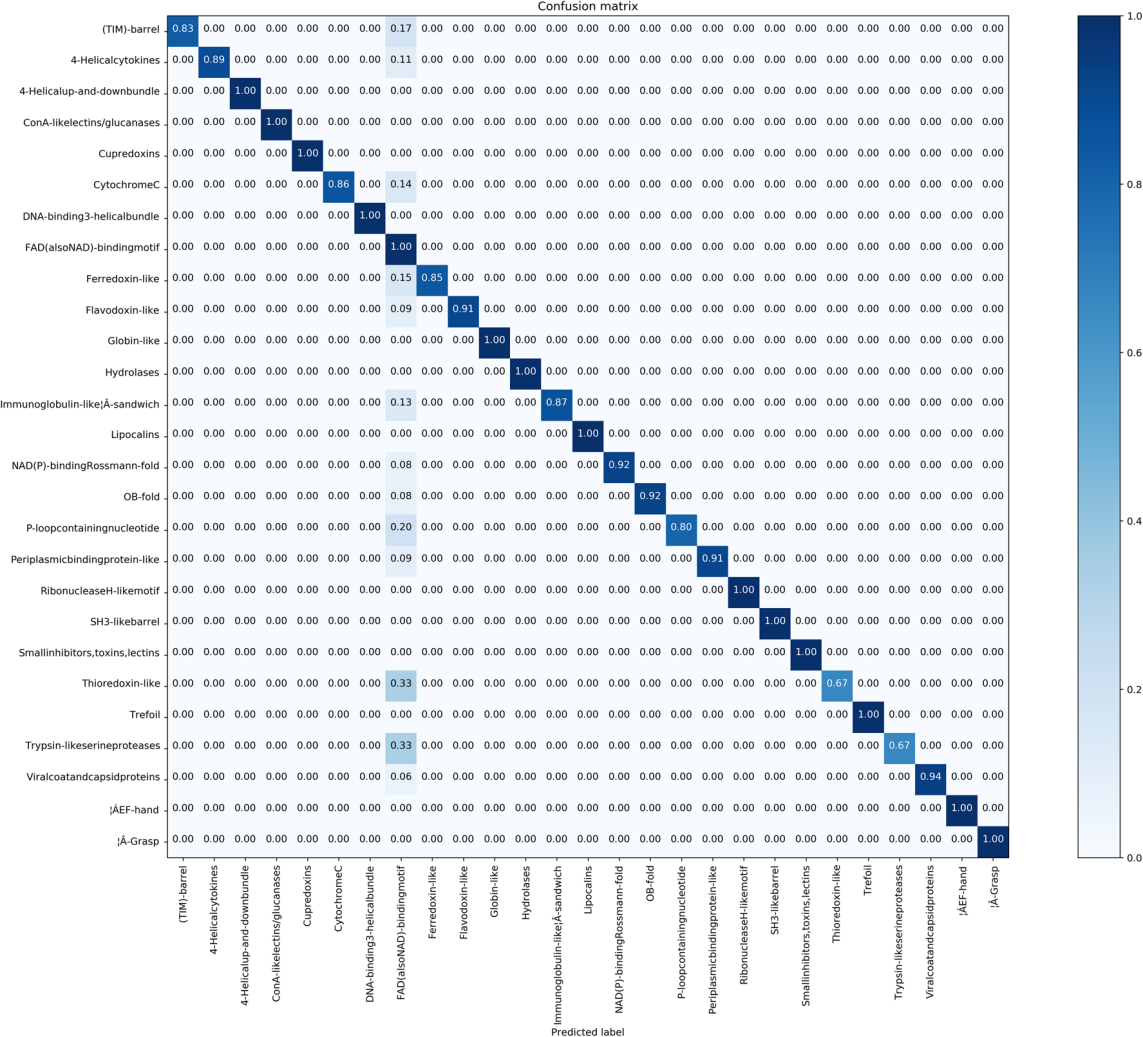
Confusion matrix of RDD dataset ($91.64\%$).

**Figure 9:**
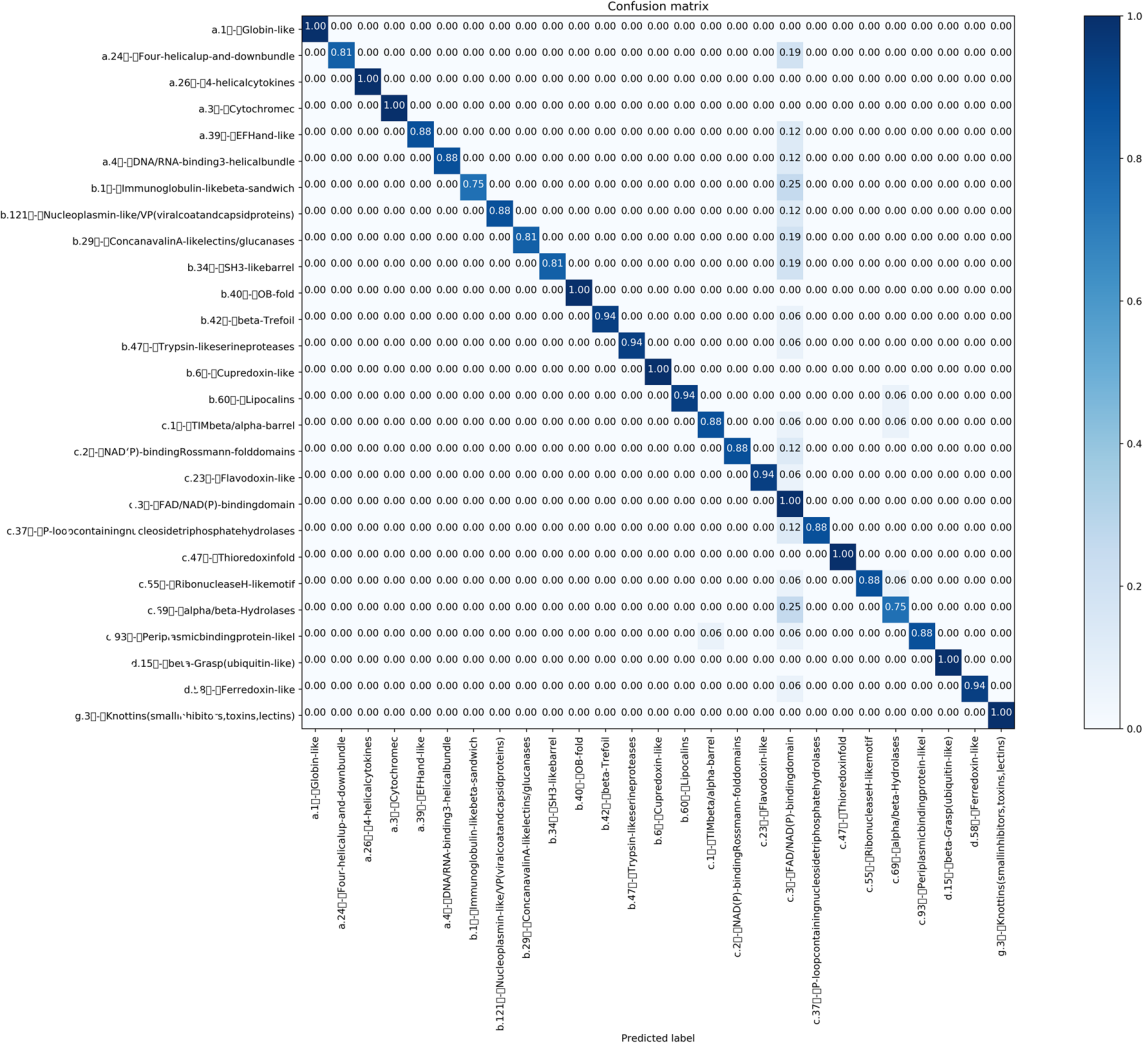
Confusion matrix of EDD dataset ($91.2\%$).

It is evident in Figs. 7, 8, and also Fig. 9, only “FAD-BINDING MOTIF” protein fold is not well recognized. To further comparative analysis, we compare “THIOREDOXIN” with “FAD-BINDING MOTIF”. According to confusion matrixes of DD, RDD and EDD, these folds are predicted false-positive in 0.33, 0.33 and 0 respectively. The proteins of Thioredoxin fold for DD and RDD are similar in number and type but the Thioredoxins-proteins for EDD are more in number and different in type. “1EGO” and “1ABA” proteins (RDD, DD) are Glutaredoxin. Dobrovolska et al.^45^, in their studies, demonstrate that Thioredoxin Glutathione Reductase and Glutaredoxin sequences have some similarity over the entire length. Thioredoxin Reductases are flavoproteins that function as homodimers with each monomer possessing a FAD prosthetic group^46^. So we guess that the “FAD-BINDING MOTIF” has similar alignments with other folds which in turn is a result of false-positive predictions.Also, these confusion matrices show the power of proposed method for predicting the other folds in these datasets.

Although the low-dimensional features can make the model more robust, an inadequate feature will make the information provided by the features insufficient and the model can only obtain a low accuracy. When we consider the features which are ranked between $[0.85 \max _{IG}, \max _{IG}]$, the accuracy of the proposed model after 10-fold cross-validation records 86.2%, 75.5%, and 72.5% for DD, RDD, and EDD respectively. So, we get almost the optimal feature subset by testing multiple regions of ranking for each dataset. Figure 5 has been shown the result of the IG method. The maximum accuracy of classification for each dataset has been achieved when we consider ranking features higher than $\frac{1}{2}\max _{IG}$ for these datasets. The number of selected features is related by the rank of features for each dataset, so the number of features for DD, RDD, and EDD are 1300, 1416, and 900 respectively. The sensitivity, precision, and F1 score are computed for each class and then averaged over all the classes which are calculated and published in Table1

**Table 1:** F1 score, sensitivity and precision, measurement tools to evaluate the proposed method.

Data set	F1 score	Sensitivity	Precision	Number of features
DD	0.98	0.92	0.93	1300
RDD	0.98	0.92	0.93	1416
EDD	0.96	0.91	0.93	900

.

**Table 2:** Comparison of the proposed method with the existing predictor and Meta-predictors for the DD, RDD and EDD.

Methods	Reference	DD	RDD	EDD
ACC + HXPZV	^4^	42.7	NA	40.9
Occurrence	^5^	42	56.6	70.0
ACC	^10^	68.0	73.8	85.9
PF1	^7^	50.6	53.3	63.0
PF2	^7^	48.2	NA	49.9
TAXFOLD	^29^	71.5	83.2	NA
Bigram	^8^	79.3	59.6	79.9
PFPA	^23^	73.6	NA	92.6
SD	^9^	86.3	72.1	90.0
Trigram	^11^	73.4	60.0	80.0
PHMM-DP	^22^	82.7	NA	92.9
MF-SRC	^47^	78.6	NA	86.2
Enhanced-SD	^34^	90.0	75.4	93.0*
Proposed Method	–	**91.31**	**91.64**	91.2

*The evaluation method not defined in^34^ approach.

## Conclusion

This study aims to improve protein fold recognition accuracy by fusing information that are extracted from the PSSM matrix. In this approach, we use ACC and SD feature extraction methods. It was observed that the proposed technique eventuates to $6\%$ improvement for the accuracy of these three benchmark datasets.

In the future, classification can be done by combining more syntactical,physiochemical or evolutionary features. To achieve more accuracy, future studies should be concentrate on “FAD-BINDING MOTIF” protein fold that has less discriminative features in the SD and the ACC. Boosting classifier may be employed to find better solutions for protein fold recognition.
